# Dietary Montmorillonite Improves the Intestinal Mucosal Barrier and Optimizes the Intestinal Microbial Community of Weaned Piglets

**DOI:** 10.3389/fmicb.2020.593056

**Published:** 2020-11-25

**Authors:** Han Liu, Congmin Wang, Xueling Gu, Jing Zhao, Cunxi Nie, Wenju Zhang, Xi Ma

**Affiliations:** ^1^State Key Laboratory of Animal Nutrition, College of Animal Science and Technology, China Agricultural University, Beijing, China; ^2^College of Animal Science and Technology, Shihezi University, Shihezi, China; ^3^Department of Dermatology, Seventh Medical Center of Chinese PLA (People’s Liberation Army) General Hospital, Beijing, China

**Keywords:** montmorillonite, growth performance, intestinal mucosal barrier, intestinal bacteria community, weaned piglets

## Abstract

The study investigated the impact of dietary montmorillonite on the growth performance, intestinal mucosal barrier, and microbial community in weaned piglets with control group (CON) and dietary supplementation of 0.2% montmorillonite (0.2% M). Compared with the CON group, 0.2% M feed in the diet increased the average daily gain (ADG) on days 15–35 and day 1–35 and the average daily feed intake on days 1–35 (ADFI) (0.05 < *P* < 0.1). Besides, higher villus height of the duodenum and jejunum and lower crypt depth of duodenum and colon were revealed in the 0.2% M group than in the CON group (*P* < 0.05). Moreover, the V/C (ratio of the villus height and crypt depth) in the 0.2% M group was increased compared to that in the CON group both from the duodenum and ileum (*P* < 0.05). The relative mRNA expression of *mucin-1*, *ITGB1* (*β1-integrins*), and *PKC* (*protein kinase C*) of ileum in the 0.2% M group were upregulated (*P* < 0.05) compared to that in the CON group. The digesta sample of ileum from piglets in the 0.2% M group contained greater (*P* < 0.05) intestinal bacterial diversity and abundances of probiotics, such as *Streptococcus*, *Eubacterium_rectale_group*, and *Lactobacillus*, which could promote the synthesis of carbon-containing biomolecules. Overall, dietary supplementation of 0.2% M was shown to have a tendency to improve the growth performance of weaned piglets and may enhance their intestinal mucosal barrier function via altering the gut microbiota.

## Introduction

Ensuring that weaned piglets can grow healthily into the fattening stage is a key issue within pig breeding that has an effect on the economic benefit of the pig breeding industry. The intestinal nutrient metabolism during the weaning period undergoes a transition from liquid milk digestion to feed digestion (Wijtten et al., 2011). This process is prone to diseases, such as bacterial diarrhea (Heo et al., 2012; Huang et al., 2015) or viral gastroenteritis (Wang Q. et al., 2019), and even death, which may be caused by the impaired intestinal mucosal barrier function (Han et al., 2016; Ma et al., 2017). A healthy and efficient intestinal mucosal barrier and microbial community in weaned piglets can contribute to a safe and rapid transition for weaned piglets to the fattening stage (Heo et al., 2012). The active substances that could enhance the intestinal function and optimize intestinal microbes are therefore a powerful weapon with which to improve the gut health and growth performance of weaned piglets.

Montmorillonite, a layered silicate, possesses various physical and chemical properties, such as adsorption and swelling (Bhattacharyya and Gupta, 2008). It was also found to include anti-bacterial properties (Williams, 2008), enhancing immunity (Lee et al., 2015), and the intestinal mucosal barrier (Das et al., 2015). Recently, many studies (Song et al., 2012; Subramaniam and Kim, 2015) have reported that the montmorillonite has the potential to improve the intestinal barrier function, digestibility of nutrients, and growth performance of weaned piglets, which may be related to the changed gut microbiota (Xia et al., 2005; Wang et al., 2012). There has been, however, limited knowledge of the interactions between montmorillonite, the intestinal barrier, and intestinal bacteria community. In the present study, the growth performance, intestinal barrier function, and intestinal bacteria community of weaned piglets were investigated in terms of the efficacy of a supplement of 0.2% montmorillonite in order to provide a theory for rational and efficient utilization of montmorillonite as a feed supplement.

## Materials and Methods

All experimental operations and processing used in this work were approved by the Animal Care and Use Committee of China Agricultural University (Beijing, China). The montmorillonite used in this work (>95% purity) was obtained from Sanding Technology Company (Zhejiang, China).

### Animal Treatment and Experimental Design

A total of 60 crossbred piglets (Duroc × Landrace × Large White) weaned at 28 ± 1 day of age, with an average initial body weight of 6.5 ± 0.5 kg, were randomly allocated to two dietary groups receiving a corn-soybean meal basal diet (CON group) or a corn-soybean meal basal diet supplemented with 0.2% montmorillonite (0.2% M) according to weight, sex, and litter with six replicates (pens) of five piglets. Feed and water were available *ad libitum* throughout the 35 days feeding trial. The basal diet was formulated to meet the nutrient requirements recommended by the NRC. The ingredient composition and nutrient content of basal diet was given in Table 1. Diets were mixed for sample and grinded to pass through a 0.15 mm sieve. Dry matter, gross energy, crude protein, calcium and total phosphorus, ether extract, and ingredient contents of the basal diets were calculated according to the procedures of the Association of Official Analytical Chemists (AOAC) International. The body weight (BW) of piglets and feed were measured individually at the beginning, end of first 2 weeks, and end of the whole experiment to calculate the average daily gain (ADG), average daily feed intake (ADFI), and feed:gain (F:G) ratio.

**TABLE 1:** The ingredient composition and nutrient content of diets (%, as-fed basis)^*a*^.

Items	Content (%)	Nutrient levels	
**Ingredient**		GE (MJ/kg)	16.95
Extruded maize meal	54.19	DM (%)	91.41
Dehulled soybean meal	20.70	CP (%)	20.26
Extruded soybean	11.00	EE (%)	8.11
Whey power	4.00	Ca (%)	0.87
Fish meal	3.00	Total P (%)	0.71
Wheat bran	1.50		
Dicalcium phosphate	2.20		
Glucose	1.00		
Limestone	0.80		
L-Lysine⋅HCl	0.35		
L-Threonine	0.18		
DL-Methionine	0.05		
Tryptophan	0.03		
Premix	1.00		
Total	100		

**^*a*^Data are the means of three replicates of analyzed values. GE, Gross Energy; DM, Dry Matter; CP, Crude Protein; EE, Ether Extract; Ca, Calcium; P, Phosphorus. Premix supplied per kg diet: vitamin A, 9,000 IU; vitamin D3, 3,000 IU; vitamin E, 20 IU; vitamin K3, 3 mg; vitamin B12, 0.2 mg; niacin, 30 mg; pantothenic acid, 15.0 mg; choline chloride, 400 mg; Zn, 75 mg; Mn, 60 mg; Fe, 75 mg; Cu, 150 mg; I, 0.35 mg; Se, 0.30 mg.**

### Sample Collection

At the end of the experiment, six piglets closest to the average BW from each group (one pig per pen) were slaughtered after being fasted overnight (12 h), and the duodenum, jejunum, ileum, and colon were then sampled through a sterile laparotomy and placed in neutral formalin for histological analysis or collected in centrifuge tubes and then immediately placed in liquid nitrogen and stored at the temperature of −80°C for analysis of mRNA expression levels of *β-actin*, *mucin-1* (*MUCIN-1*), *β1-integrins* (*ITGB1*), *collagen*, *occludin*, and *protein kinase C* (*PKC*) in ileal tissue. Briefly, about 1 cm length of the middle part of duodenum, jejunum, ileum, and colon were collected. The digesta of ileum and colon were obtained using centrifuge tubes and immediately placed in liquid nitrogen and then stored at the temperature of −80°C for analysis of the bacterial community.

### Intestinal Morphology Analysis, RNA Extraction, and Quantitative RT-PCR Analysis

Samples from duodenal, jejunal, ileal, and colonic segment were embedded in paraffin and cut into 5 μm sections. Six non-successive sections of each sample were identified with hematoxylin and eosin staining. Six well-oriented villi and their associated crypt per section from each sample were collected. The villus height and crypt depth of duodenum, jejunum, ileum, and colon were measured and analyzed using a Leica Image Processing with Analysis System (Leica Imaging Systems Limited, Berlin, Germany). Total RNA extraction and quantitative real-time PCR analysis were conducted as described previously (Shen et al., 2014) with modifications. Briefly, TRIzol reagent (Invitrogen, Carlsbad, United States) was used to extract the total RNA of ileum after tissue homogenization and mixed with DNase I (Invitrogen, Carlsbad, United States). The obtained total RNA of IM (ileum with 0.2% montmorillonite) and INC (ileum negative control) were checked by 1% agarose gel electrophoresis and 2100 Bioanalyzer RNA Nanochip (Agilent, Palo Alto, United States). PrimeScript^TM^ RT Reagent Kit (Takara, Dalian, China) was used to perform the reverse transcription of total RNA. Expression levels of *β-actin*, *MUCIN-1*, *ITGB1*, *collagen*, *occludin*, and *PKC* in ileal tissue were analyzed by Roche LightCyler 480 system (Roche, Basel, Switzerland). The primer sequences for these six genes were showed in Table 2.

**TABLE 2:** Primers used for quantitative RT-PCR.

Gene	Forward (5′–3′)	Reverse (5′–3′)
β-actin	ACACGGTGCCCATCTACGAG	GCTTCTCCTTGATGTCCCGC
Occludin	CTTTCTCAGCCAGCGTATTC	AGGCAAGCGTGGAGGCAACA
Mucin-1	CGGAAGCAGGCACCTATAAC	CAGAATACAGACCAGCACCA
β1-integrins	TAAGAGTGCCGTGACAACCG	TTCAGAACCTGCCCATAGCG
Collagen	TGCTGCTGCTATTGTCCTTG	ACTGTGCCTTGGTGTTGGAT
Protein kinase C	CTCACTGCCACAACACAACT	GCACGAGCGGTTCTTCACTG

The RT-PCR system was: 10 μL of 2 × SYBR^®^ premix Ex TaqTM II, 0.6 μL of each forward and reverse primer (10 μmol/L), 2 μL of complementary DNA template, and 6.8 μL of double distilled water. The PCR reaction included an inactivation step at 95°C for 5 min, and this was followed by 35 cycles of denaturation at 95°C for 10 s, annealing at 60°C for 10 s, and extension at 72°C for 15 s. Each reaction was conducted to 20 μL using LightCycler 480 SYBR Green 1 Master (Roche, Basel, Switzerland). Each gene was performed with triplicate biological replicates and technical replicates. The result was calculated and represented by the 2^–ΔΔ*CT*^ method.

### Intestinal Microbial DNA Extraction and High-Throughput Sequencing

Isolation of ileal and colonic content was performed using DNA Stool Mini Kit (Qiagen, Hilden, Germany). The quality and size of extracted DNA were checked via a NanoDrop 2000 spectrophotometer (Thermo Fisher Scientific, Waltham, United States) and 1% agarose gel electrophoresis. The quantified DNA was stored in −20°C for next analysis. Amplification of V3–V4 regions of the bacterial 16S rRNA gene was performed by TransStart FastpfuR DNA Polymerase (Takara, Dalian, China). The upstream primer and the downstream primer were 5′-barcode-ACTCCTACGGGAGGCAGCA-3′ and 5′-GGACTACHVGGGTWTCTAAT-3′, respectively. Amplification PCR reactions was conducted in 20 μL, with 10 ng template DNA, 1U FastPfu polymerase, 1 × FastPfu buffer, 250 μM dNTP, and 0.1 μM each of the primer. PCR was under the reaction conditions of 95°C for 2 min, 95°C for 30 s, 55°C for 30 s, 72°C for 30 s for 30 cycles, and then 72°C for 5 min. PCR products were firstly purified using AxyPrep DNA Purification kit (Axygen Biosciences, Union City, United States) after being run through 2% agarose gel electrophoresis. On agarose gels, the PCR products were quantified by QuantiFluor-ST Fluorimeter (Promega, Wisconsin, United States) using a PicoGreen dsDNA Quantitation Kit (Invitrogen, Carlsbad, United States). Purified amplicons were gathered in equimolar ratios for 2 × 300 bp sequencing by Illumina MiSeq in Shanghai Majorbio Bio-pharm Technology Co., Ltd. (Beijing, China) based on the standard protocols. Each treatment group was performed six replications.

### Bioinformatics Analysis of Sequencing Reads

QIIME (version 1.9.1) and Fastp (version 0.19.6) were used for quality control and sequence filtering with the following criteria: (1) sequencing reads were clipped with an average quality score of < 20; (2) reads shorter than 50 bp were dropped; (3) reads with two nucleotides of mismatch in primer sequences or ambiguous nucleotides were deleted; and (4) paired reads with less than 10 bp overlap were discarded. Operational taxonomic units (OTUs) with a 97% identity cutoff were gathered by Uparse (version 7.0.1090)^1^, and the analysis of taxonomy of OTUs was performed using the Silva (Release132)^2^ 16S rRNA database. The α-diversity indices, including Chao 1 and Shannon, were analyzed by Mothur v.1.30.2. PCoA tools in R language were used for principal co-ordinates analysis (PCoA). The histogram of linear discriminant analysis (LDA) distribution was implemented using LDA effect size analysis (LEfSe) software. The 16S rRNA gene sequencing information was analyzed by PICRUSt to predict biological functions (EggNOG database)^3^ and metabolic pathways (KEGG database)^4^ of the bacterial community of ileal and colonic contents samples of weaned piglets.

### Statistical Analysis

The data (growth performance, intestinal morphology, and quantitative RT-PCR) analysis was performed by unpaired *t*-test of SPSS 19.0. Results are represented as means ± SEM. The data (α-diversity indices, predictive analysis of metabolic functions, and metabolic pathways) analysis was performed via a Wilcoxon rank-sum test. LDA analysis was performed by non-parametric factorial Kruskal-Wallis (KW) sum-rank test. *P*-value < 0.05 was considered as statistically significant.

## Results

### Growth Performance

The results shown that there are no significantly difference in the ADG, ADFI and F:G (*P* > 0.05) of weaned piglets no matter what period during the whole experiment (Table 3). However, the 0.2% M has shown a tendency to improve the ADG during days 15–35 and 1–35 as well as the ADFI during days 1–35 (0.05 < *P* < 0.1).

**TABLE 3:** Effects of dietary supplement with 0.2% montmorillonite on growth performance in weaned piglets^*a*^.

Item	CON	0.2% M	*P*-value
**1–14 days**			
ADG	227 ± 28.3	223.9 ± 17.7	0.25
ADFI	493.5 ± 30.7	474.6 ± 32.6	0.18
F:G	2.17 ± 0.1	2.12 ± 0.3	0.13
**15–35 days**			
ADG	478 ± 28.9	495.4 ± 23.9	0.09
ADFI	1057.9 ± 30.5	1000.3 ± 34.7	0.11
F:G	2.22 ± 0.1	2.02 ± 0.1	0.15
**1–35 days**			
ADG	377.5 ± 16.9	386.8 ± 20.9	0.07
ADFI	832.4 ± 25.8	790.2 ± 40.5	0.09
F:G	2.21 ± 0.2	2.04 ± 0.3	0.13

**^*a*^Data were shown as the mean ± SEM (n = 30). CON, basic diet control; 0.2% M, basic diet with 0.2% montmorillonite group; ADFI, average daily feed intake; ADG, average daily gain; F/G, ratio of ADFI/ADG.**

### Intestinal Mucosal Morphology

**FIGURE 1:**
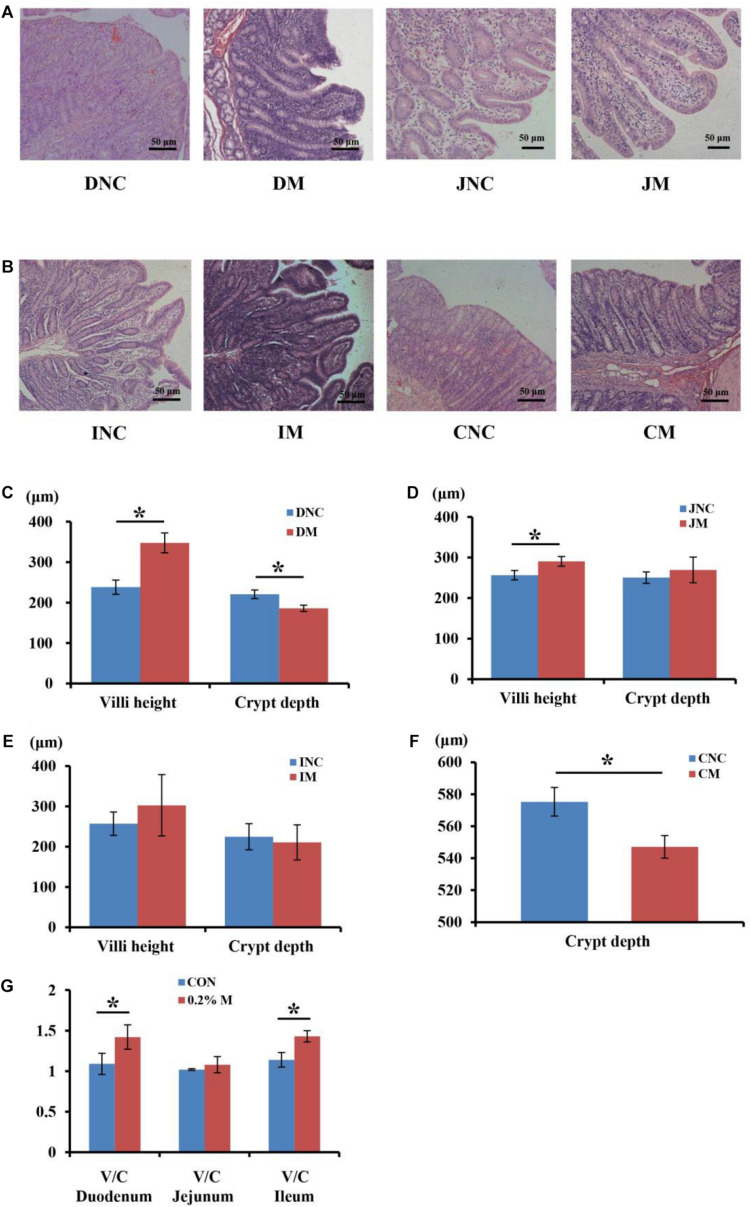
Effects of 0.2% montmorillonite on the small intestine and colon mucosal morphology. **(A)** Mucosal morphology of duodenum and jejunum from weaned piglets. **(B)** Mucosal morphology of ileum and colon from weaned piglets. The changes of villus height and crypt depth of **(C)** duodenum, **(D)** jejunum, and **(E)** ileum were investigated. **(F)** The changes of crypt depth of colon. **(G)** The ratio of the villus height and crypt depth (V/C) of duodenum, jejunum and ileum. Values are means ± SEM, *n* = 6. *P < 0.05. DNC, duodenum negative control; DM, duodenum with 0.2% montmorillonite; JNC, jejunum negative control; JM, jejunum with 0.2% montmorillonite; INC, ileum negative control; IM, Ileum with 0.2% montmorillonite; CNC, colon negative control; CM, colon with 0.2% montmorillonite.

The mucosal morphology of the duodenum, jejunum, ileum, and colon were obtained in Figure 1. The villus height of DM (duodenum with 0.2% montmorillonite) and JM (jejunum with 0.2% montmorillonite) was significantly higher (*P* < 0.05) than that in DNC (duodenum negative control) and JNC (jejunum negative control), respectively (Figures 1C,D). Conversely, the crypt depth in DM and CM (colon with 0.2% montmorillonite) was significantly lower (*P* < 0.05) than that in DNC and CNC (colon negative control), respectively (Figures 1C,F). The differences of the villus height and crypt depth (Figure 1E) between IM and INC were non-significant, respectively (*P* > 0.05). Besides, the V/C (ratio of the villus height and crypt depth) in the 0.2% M group was significantly increased (*P* < 0.05) in comparison to that in the CON group for both the duodenum and ileum (Figure 1G).

### Intestinal Barrier Function

**FIGURE 2:**
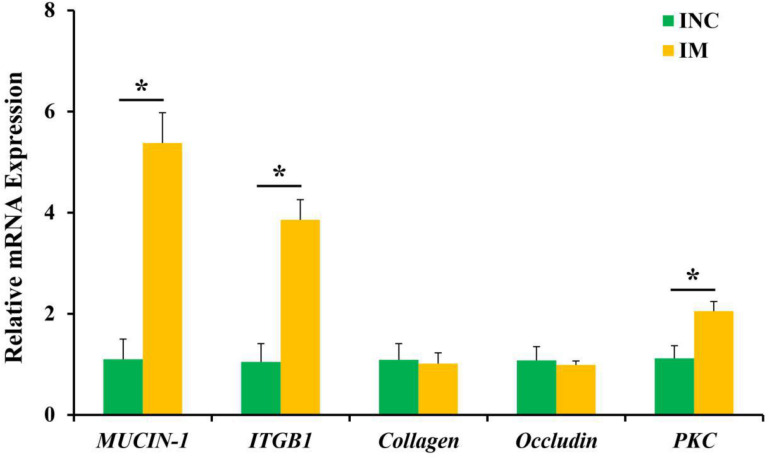
Effects of 0.2% montmorillonite on the relative mRNA expressions of ileal barrier functional gene. Bate-actin was used as an internal standard for normalization. Values are means ± SEM for three independent biological and technical replications, *n* = 6. *P < 0.05. ITGB1, β1-integrin; PKC, protein kinase C.

The relative mRNA expression of ileal barrier function-related genes is displayed in Figure 2. A diet containing 0.2% M has been shown to upregulate (*P* < 0.05) the relative mRNA expression of *MUCIN-1*, *ITGB1*, and *PKC* in the ileum of piglets (Figure 2). However, the relative mRNA expressions of *collagen* and *occludin* have shown no significantly difference (*P* > 0.05) between the CON and 0.2% M groups in the ileum of piglets (Figure 2).

### The Bacterial Community of Digesta From the Ileum and Colon

**FIGURE 3:**
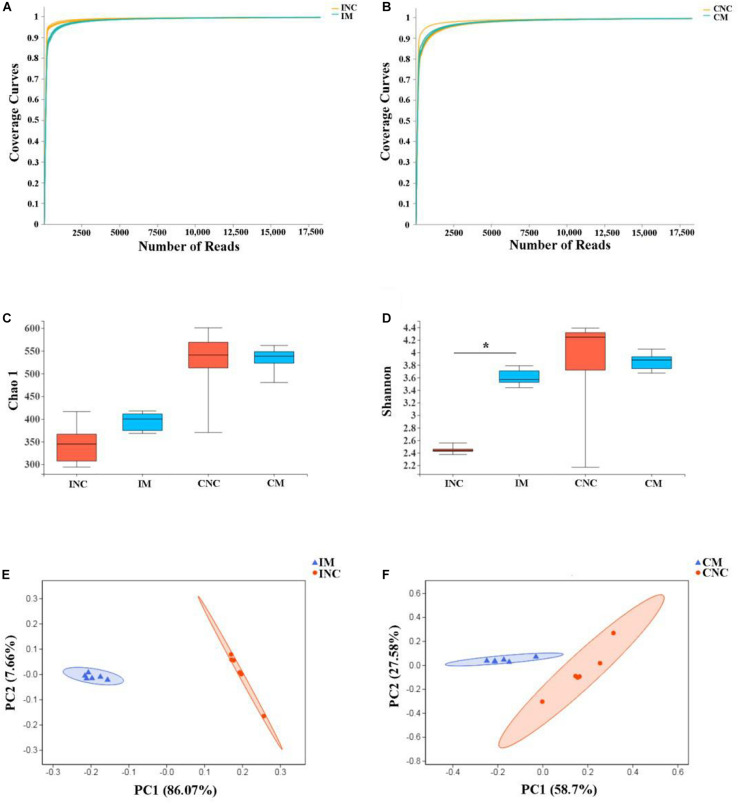
Alpha diversity and PCoA of bacterial communities between control and montmorillonite treatments in ileum and colon from weaned piglets. Sequencing coverage curves of negative control (INC) and **(A)** basal diet with 0.2% montmorillonite (IS) from the ileum and negative control (CNC) and **(B)** basal diet with 0.2% montmorillonite (CS) from the colon. **(C)** Chao 1 index of bacterial community and **(D)** Shannon index of bacterial community in four groups. The PCoA analyses of bacterial communities between control and montmorillonite treatment group from **(E)** ileum and **(F)** colon. The results were analyzed by Wilcoxon rank-sum test and *P < 0.05.

To investigate the effects of montmorillonite on the bacterial community structure of weaned piglets, we performed 16S rRNA gene sequencing of content samples from the ileum and colon. After sequence quality control, a total of 470445 as well as 424655 clean reads (Table 4) were obtained from the CON and 0.2% M group, respectively. The OTUs numbers of INC, CNC, IM, and CM with six biological replications are also listed in Table 4. Based on OTUs information, bioinformatics analysis was further performed. The coverage curves (Figures 3A,B) showed flat trend with the increasing of sequencing reads, indicating that the sequencing reads in this experiment were sufficient to reveal the bacterial diversity of content samples from ileum and colon. Besides, the Shannon index in IM was higher (*P* < 0.05) than that in INC (Figure 3D), while there were no significantly difference in the Chao 1 index of the ileum and colon and the Shannon index of the colon between the CON and 0.2% M groups (Figures 3C,D). PCoA analysis (Figures 3E,F) showed a clear differentiation (*P*_*IM*__–__*INC*_< 0.05, *P*_*CM*__–__*CNC*_< 0.05) between the CON and 0.2% M groups both in the microbiota present in ileum and colon, indicating that the addition of montmorillonite changed the bacterial community structure in ileum and colon.

**TABLE 4:** Statistics of bacterial 16S rRNA gene amplicon sequencing for ileal and colonic content^*a*^.

Group ID	Clean reads	Average length (bp)	OTUs
INC1	48,572	424.62	186
INC2	61,439	413.79	250
INC3	25,883	416.95	233
INC4	47,961	415.08	248
INC5	34,086	417.09	217
INC6	34,817	414.54	251
CNC1	36,927	420.27	399
CNC2	21,440	417.53	508
CNC3	45,335	415.20	466
CNC4	34,634	417.17	483
CNC5	54,390	416.75	465
CNC6	24,961	425.23	266
IM1	35,696	435.53	297
IM2	30,865	433.52	314
IM3	34,132	435.96	303
IM4	34,666	433.90	346
IM5	31,539	433.92	333
IM6	33,761	433.66	307
CM1	33,011	431.03	455
CM2	44,915	432.13	457
CM3	32,829	430.11	447
CM4	36,228	431.58	400
CM5	39,203	431.15	459
CM6	37,810	434.97	411

**^*a*^INC, Ileum negative control; CNC, Colon negative control; IM, Ileum with 0.2% montmorillonite; CM, Colon with 0.2% montmorillonite. Sequences with similarity scores ≥ 0.97 were clustered into an OTU.**

**FIGURE 4:**
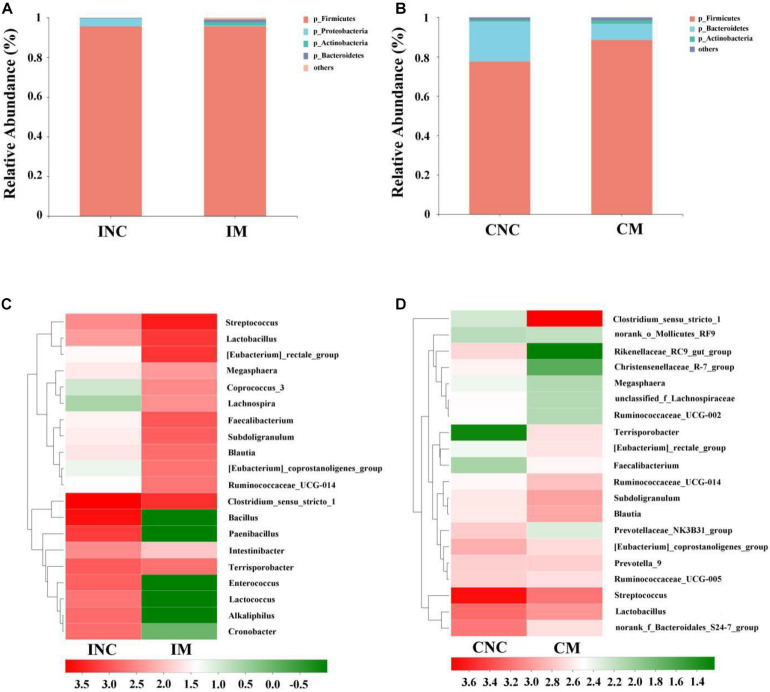
Bacterial diversity at the phylum and genus level in the ileum and colon from weaned piglets of control and treatment group. **(A)** Bacterial community barplot of INC and IM at the phylum level in ileum. **(B)** Bacterial community barplot of CNC and CM at the phylum level in colon. **(C)** Bacterial community barplot of INC and IM at the genus level in ileum. **(D)** Bacterial community barplot of CNC and CM at the genus level in colon. INC, ileum negative control; CNC, colon negative control; IM, ileum with 0.2% montmorillonite; CM, colon with 0.2% montmorillonite.

At the phylum level, the relative abundance of Firmicutes in IM and INC was slightly changed (95.7 vs. 95.6%), and the relative abundance of Firmicutes in CM (77.6 vs. 88.5%) was enhanced compared to CNC. The relative abundance of Actinobacteria in IM (0.1 vs. 1.6%) and CM (0.8 vs. 1.6%) was enhanced (Figure 4A), while the Proteobacteria in IM (4.0 vs. 0.4%) and the Bacteroidetes in CM (20.3 vs. 8.3%) was reduced compared to that in INC an CNC (Figure 4B), respectively. At the genera level, the relative abundance of the top 20 bacterial communities in INC, IM, CNC, and CM is listed in the heatmap (Figures 4C,D). The relative abundance of *Clostridium_sensu_stricto_1*, *Bacillus*, *Paenibacillus*, and *Terrisporobacter*, the dominant genera in INC, was decreased in IM (36.7 vs. 15.7%; 27.3 vs. <0.01%; 10.1 vs. <0.01%; 5.1 vs. 3.7%), and the dominant genera in IM changed to *Streptococcus* (24.2%), *Clostridium_sensu_stricto_1* (15.7%), *[Eubacterium_rectale_group* (13.8%), and *Lactobacillus* (13.2%), which was richer than that in INC (Figure 4C). In the colon, the dominant genera including *Streptococcus* (35.3%), *Lactobacillus* (12.1%), *norank_f_Bacteroidales_S24-7_group* (10.4%), and *[Eubacterium]_coprostanoligenes_group* (5.5%) in CNC changed to *Clostridium_sensu_stricto_1* (39.5%), *Streptococcus* (10.3%), and *Lactobacillus* (7.1%) in CM (Figure 4D). In addition, the relative abundance of *Clostridium_sensu_stricto_1*, *Terrisporobacter*, *Subdoligranulum*, and *Blautia* dramatically increased, but the *Streptococcus*, *Rikenellaceae_RC9_gut_group* and *Christensenellaceae_R-7_group* reduced in CM in comparison to CNC (Figure 4D).

**FIGURE 5:**
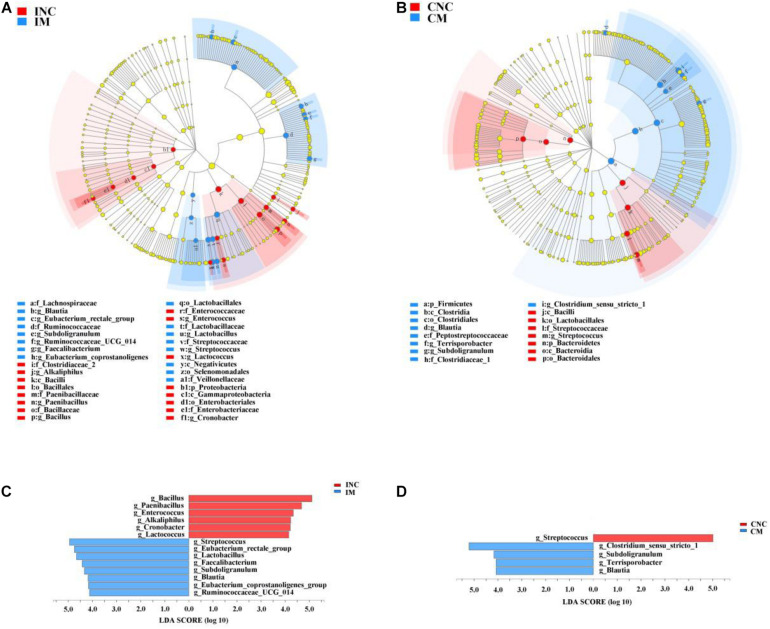
LEfSe analysis showed significantly changed (*P* < 0.01) bacteria between control and treatment group in the ileum and colon. **(A)** Significantly changed bacteria between INC and IS from phylum to genus levels. **(B)** Significantly changed bacteria between CNC and CM from phylum to genus levels. **(C)** LDA bar chart showed significantly changed bacteria at genus level between INC and IM. **(D)** LDA bar chart showed significantly changed bacteria at genus level between CNC and CM. LDA score > 4 as the cutoff value. INC, ileum negative control; CNC, colon negative control; IS, ileum with 0.2% montmorillonite; CM, colon with 0.2% montmorillonite.

All differential bacteria of the ileum and colon were demonstrated from the phylum to species level in cladogram of LEfSe between CON and 0.2% M groups (Figures 5A,B). At the phylum level, the relative abundance of Proteobacteria in the ileum and the Bacteroidetes in the colon were significantly lower (*P* < 0.05) in 0.2% M group than that in CON group (Figures 5A,B). In the genera level, the relative abundance of *Bacillus*, *Paenibacillus*, *Enterococcus*, *Alkaliphilus*, *Cronobacter*, and *Lactococcus* were markedly abated (*P* < 0.05), while the relative abundance of *Streptococcus*, *Eubacterium_rectale_group*, *Lactobacillus*, *Faetobacterium*, *Subdoligranulum*, *Blautia*, *Eubacterium_coprostanoligenes_group*, and *Ruminococcaceae_UCG_014* were signally boosted (*P* < 0.05) in IM instead of INC (Figure 5C). Moreover, the relative abundance of *Streptococcus* were dramatically reduced (*P* < 0.05), but the relative abundance of *Clostridium_sensu_stricto_1*, *Subdoligranulum*, *Terrisporobacter*, and *Blautia* were observably enhanced (*P* < 0.05) in CM instead of CNC (Figure 5D).

### Predicted Functional Profiles of Microbial Communities Using PICRUSt

**FIGURE 6:**
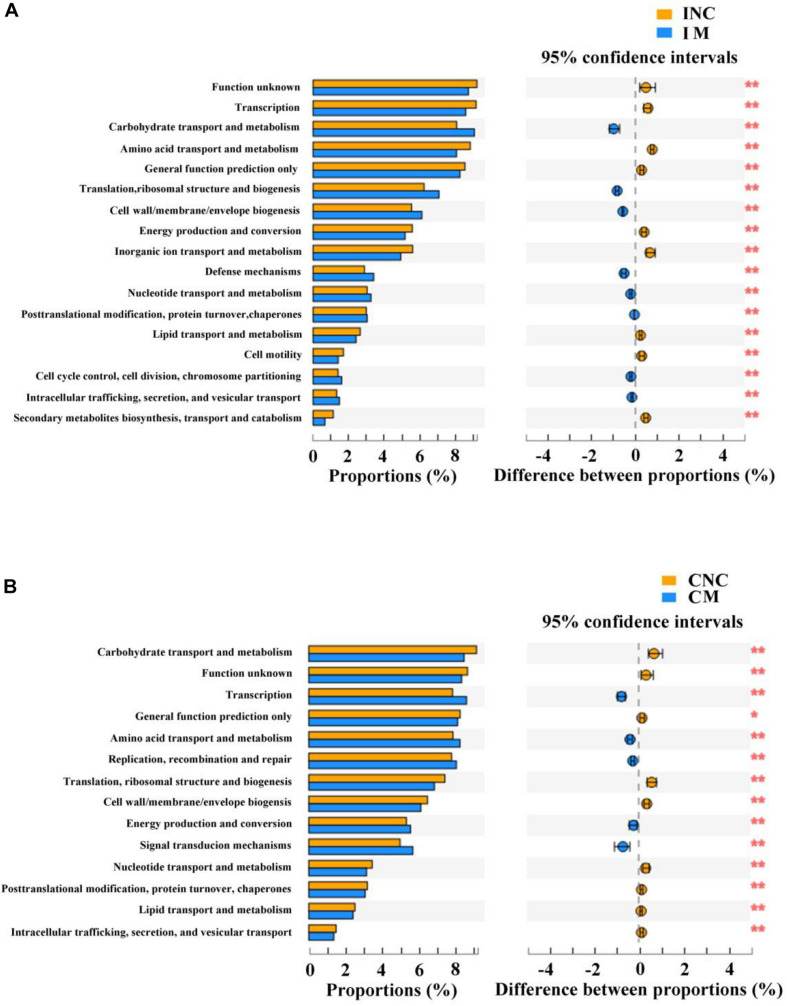
Comparison of the bacterial predicted biological functions (COG level 1) between control and treatment group from the ileum and colon using PICRUSt. **(A)** COG function prediction analysis of ileal bacteria between INC and IM. **(B)** COG function prediction analysis of colonic bacteria between CNC and CM. INC, ileum negative control; CNC, colon negative control; IM, ileum with 0.2% montmorillonite; CM, colon with 0.2% montmorillonite. *P < 0.05 and **P < 0.01.

**FIGURE 7:**
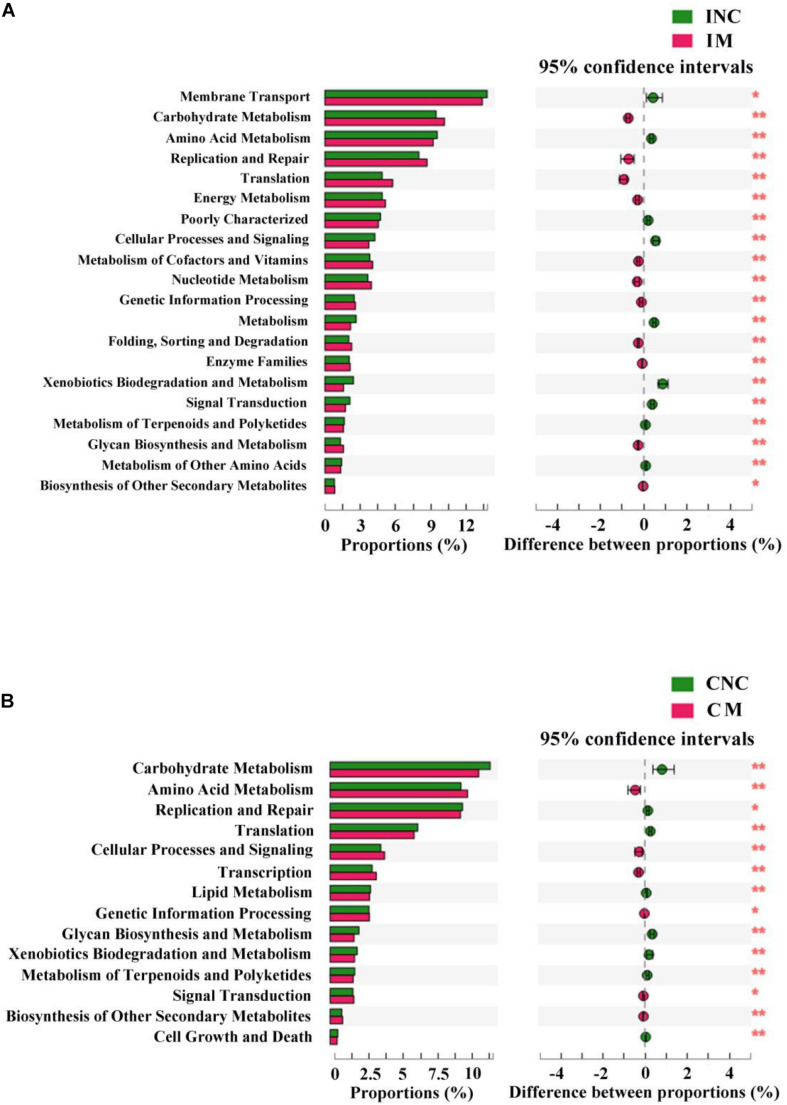
The mean proportion and significant difference in predicted metabolism pathways (KEGG) between control and treatment group from the ileum and colon using PICRUSt. **(A)** KEGG pathways analysis of ileal bacteria between INC and IM. **(B)** KEGG pathways analysis of colonic bacteria between CNC and CM. INC, ileum negative control; CNC, colon negative control; IM, ileum with 0.2% montmorillonite; CM, colon with 0.2% montmorillonite. *P < 0.05 and **P < 0.01.

To predict the potential function of gut bacteria on nutrient metabolism in piglets after feeding on a diet with and without the montmorillonite, biological functions and KEGG pathways were analyzed by the PICRUSt program. Seventeen notably different predicted biological functions (COG level 1) and 20 markedly different KEGG pathways between INC and IM and 14 different predicted biological functions and 14 different KEGG pathways between CNC and CM were detected in Figures 6, 7. Beyond this, bacteria with carbohydrate transport and metabolism, translation, ribosomal structure and biogenesis, cell wall/membrane/envelope biogenesis, defense mechanisms, and nucleotide transport and metabolism functions were significantly more enriched (*P* < 0.01) in IM than in INC (Figure 6A). The predicted metabolic pathways involved in microorganisms, such as carbohydrate metabolism, replication and repair, translation, energy metabolism, and nucleotide metabolism, were significantly enriched (*P* < 0.01) in IM compared to INC (Figure 7A). In the colon, significantly increased bacteria after dietary supplementation of 0.2% montmorillonite improved (*P* < 0.01) the functions of transcription, amino acid transport and metabolism, replication, recombination and repair, and signal transduction mechanisms (Figure 6B). The number of significantly changed predicted metabolic pathways between CNC and CM were reduced. In CM (Figure 7B), amino acid metabolism, cellular processes and signaling, and transcription were significantly enriched (*P* < 0.01).

## Discussion

Montmorillonite is widely used as a feed additive, and its characteristics of improving the growth performance of animals have attracted much attention (Yu et al., 2008; Duan et al., 2013; Yang et al., 2014; Jiao et al., 2015, 2018). Yu et al. (2008) reported that the addition of 0.5% montmorillonite had significantly improved the weight gain, feed intake, and feed conversion ratio of pigs. However, in the present study, the ADG, ADFI, and F:G of weaned piglets were not significantly changed after the addition of 0.2% montmorillonite except for an improvement trend in ADG and ADFI during days 15–35 and 1–35, respectively. Similarly with our study, the addition of graded concentration of montmorillonite only changed the feed intake without improving the weight gain of pigs (Duan et al., 2013). It is also reported that adding montmorillonite to the feed did not improve the growth performance of pigs (Xia et al., 2005; Song et al., 2012). Interestingly, relying on the adsorption of montmorillonite, feeding montmorillonite loaded with copper and zinc ions, which are beneficial to growth, showed a significant improvement in the growth performance of pigs (Jiao et al., 2015, 2018); this is an indication that the single addition of montmorillonite has a limited effect on improving the growth performance of weaned piglets.

Although the superior growth performance of weaned piglets was not achieved after adding the montmorillonite to the diet, it has the potential to improve the intestinal barrier function (Song et al., 2012; Subramaniam and Kim, 2015). Intestinal mucosal barriers include physical barriers, chemical barriers, immune barriers, and microbial barriers, while intestinal mucosal morphology involves the function of intestinal physical barrier. The short villi not only causes an increase in the intestinal epithelial permeability, increasing the inflammatory response and amplifying the dysfunction of the intestinal motor function, but also leads to a decrease in the intestinal absorption area, which may affect the normal intake of piglets (Collins, 2001; Liu et al., 2017). Besides, the crypt is involved in the generation and transportation of epithelial cells, and the decrease of crypt depth can increase the rate of mature cells (Clevers, 2013; Liu et al., 2014). In the present study, we found that the weaned piglets fed with the 0.2% montmorillonite diet exhibited a higher villus height in the duodenum and jejunum compared with the CON group. Meanwhile, lower crypt depth of the duodenum and colon were revealed in the 0.2% M group than in the CON group, and the V/C in the 0.2% M group was significantly increased in comparison to that in the CON group for both the duodenum and ileum. These results hinted that the montmorillonite could improve the surface area for nutrient absorption, thus increasing nutrient digestibility (Clevers, 2013; Liu et al., 2014) as well as increase the intestinal defense function by improving the intestinal mucosal morphology, which may be relate to the altering of the gut microbiota (Xia et al., 2004; Shameli et al., 2011; Placha et al., 2014).

To further confirm the effect of montmorillonite on intestinal barrier function, the relative mRNA expression of intestinal mucosal barrier-related gene (*MUCIN-1*, *ITGB1*, *collagen*, *occluding*, and *PKC*) from the ileum were measured by qRT-PCR. The results shown that the relative mRNA expression of ileal *MUCIN-1*, *ITGB1*, and *PKC* were upregulated in the 0.2% M group vs. the CON group, while the relative mRNA expression of ileac *collagen* and *occludin* have no significantly difference between the two groups. *Mucins* are mainly involved in maintaining the structure and function of intestinal mucosa and regulating the intestinal microorganisms (Mcguckin et al., 2011). *Integrins*, including *ITGB1*, are the major receptors for extracellular matrix, and regulate the assembly of adhesive junctions in the intestinal tract, and then play roles in the rapid renewal and digestive functions of intestinal tract (Breau et al., 2009). *PKC* is another important protein that is expressed by gastrointestinal cells and regulates intracellular signaling and barrier integrity (Farhadi, 2005). That the relative mRNA expression of ileal *MUCIN-1*, *ITGB1*, and *PKC* were upregulated by adding montmorillonite revealed that the addition of montmorillonite improved the intestinal barrier function (Hu et al., 2012; Jiao et al., 2015, 2018; Chen J. F. et al., 2019, 2020); this was caused by the swelling property of montmorillonite, which will cause the volume of intake feed to become larger, slowing down the time of passing through the intestinal tract and promoting the metabolism of intestinal epidermal cells (Subramaniam and Kim, 2015).

Gut microbiota, serving as microbial barriers in intestinal mucosal barriers, play an important role in intestinal function; these functions involve nutrient absorption, mucosal barrier homeostasis, immunomodulation, and defense against pathogens for pigs (Huang et al., 2015; Fan et al., 2017). It is reported that the montmorillonite improved the intestinal barrier function, digestibility of nutrients, and growth performance of weaned piglets by regulating the gut microbiota (Xia et al., 2005; Wang et al., 2012). In the present study, the Shannon index, one of the α-diversity indices, of ileum was more increased in the 0.2% M group compared with the CON group, which indicated that the addition of 0.2% montmorillonite to the feed promoted the growth of ileal bacteria in weaned piglets and improved the diversity of the microbial community. The bacterial diversity is an indicator of a healthy and stable gut microbial community, which is beneficial to the host (Salonen et al., 2012). Besides, the results of PCoA analyses proved that there is a significant difference in the microbial community composition between the CON and 0.2% M groups, which was demonstrated in that the structure and composition of gut microbiota on weaned piglets will be changed by the montmorillonite.

At the phylum level, the 0.2% M group saw a decreased relative abundance of Proteobacteria in iluem compared to the CON group in the present study. The Proteobacteria are a minor constituent within a balanced gut-associated microbial community (Eckburg et al., 2005). Recent studies have proposed that an expansion of the Proteobacteria could be a potential diagnostic microbial signature of epithelial dysfunction as well as dysbiosis in the gut microbiota (Na-Ri et al., 2015). Besides, the relative abundance of Bacteroidetes was decreased, but the Firmicutes was increased in the colon after supplementing the montmorillonite into the diets of weaned piglets. The Firmicutes and Bacteroidetes are the dominate phylum in the gut microbiota of piglets, and the increase in Firmicutes was considered a means of enhancing the body’s capacity for energy acquisition from the diet, which may improve the growth of piglets (Turnbaugh et al., 2006).

In the genera level, the relative abundance of *Streptococcus*, *Eubacterium_rectale_group*, and *Lactobacillus* in the ileum was increased by adding the montmorillonite to the diets. *Streptococcus* is considered to be involved in the process of intestinal nutrition metabolism, such as amino acid utilization (Neis et al., 2015) and the production of short-chain fatty acids (Corr et al., 2009); and butyrate belonged to short-chain fatty acids (SCFAs) contributes to maintaining intestinal homeostasis (Iacob and Iacob, 2019). In addition, the D-alanine (Miyauchi et al., 2012) and exopolysaccharide (Chen Y. et al., 2019), produced by *Streptococcus thermophilus*, and the yogurt fermented by *Streptococcus thermophilus* 1131 (Usui et al., 2018) can improve the intestinal barrier mucosal function and alleviate intestinal inflammation. Moreover, the *Eubacterium rectale* participated in the butyrogenic effect of dietary fermentation (Louis et al., 2007). The growth of *Eubacterium rectale* was inhibited in a gut model from ulcerative colitis patients, indicting *Eubacterium rectale* might promote intestinal function through butyrate metabolism (Joan et al., 2012). *Lactobacillus* is considered to be a probiotic with the function of enhancing human and animal health (Colum et al., 2001). In addition, *Lactobacillus* is believed to be involved in the production of butyrate (Berni Canani et al., 2016). As an immunoregulatory factor, butyrate maintains the intestinal barrier and immune homeostasis; meanwhile, it also promotes the release of antimicrobial peptides and de-represses microbial virulence factors against pathogen invasion (Iacob and Iacob, 2019). The increase in *Lactobacillus* might therefore further improve the intestinal metabolism and growth performance of weaned piglets in the manner of butyrate metabolism. In the colon, meanwhile, the microflora was changed slightly. For instance, the relative abundance of *Clostridium_sensu_stricto_1* and *Subdoligranulum*, which can produce SCFAs, was more enhanced in the 0.2% M group compared with the CON group, which may be due to that the montmorillonite mainly contribute to the foregut rather than hindgut (Wang L. et al., 2019). Furthermore, using the PICRUSt program to predict functional profiles of microbial communities of the ileum and colon, we found the number of gene tags involved in the synthesis of carbon-containing biomolecules in lieum and the number of gene tags involved in the energy production and conversion and the amino acid metabolism in colon were markedly more enhanced in the 0.2% M group compared with the CON group. The result suggests that (Xu et al., 2017; Lee et al., 2018) the montmorillonite supplementation may be involved in carbohydrate metabolism and amino acid metabolism by altering the gut microbiota that can product the SCFAs, but it is necessary to conduct further study into the relationship between montmorillonite supplementation and carbohydrate and amino acid metabolism.

## Conclusion

Overall, dietary supplementation of 0.2% montmorillonite has displayed a slight influence on the growth performance of weaned piglets. However, 0.2% montmorillonite supplementation improved the intestinal mucosal morphology and increased the relative mRNA expression of intestinal mucosal barrier function-related genes by altering gut microbiota. These findings will contribute to a better understanding of how montmorillonite modulates gut health through nutrient intervention.

## Data Availability Statement

The original contributions presented in the study are publicly available. This data can be found here: https://www.ncbi.nlm.nih.gov/PRJNA667820.

## Ethics Statement

The animal study was reviewed and approved by the Animal Care and Use Committee of China Agricultural University.

## Author Contributions

XM conceived and designed the research. HL, CW, and XG conducted the research. HL wrote the manuscript and analyzed the data. JZ and XG wrote a part of manuscript and assisted in analysis of data. XG and XM critically reviewed the manuscript. XG contributed to language review. All authors read and approved the final manuscript.

## Conflict of Interest

The authors declare that the research was conducted in the absence of any commercial or financial relationships that could be construed as a potential conflict of interest.

## Footnotes

1 http://drive5.com/uparse/

2 http://www.arb-silva.de

3 http://eggnog.embl.de/

4 http://www.genome.jp/kegg/

## Abbreviations

NRC: National Research Committee
AOAC: Association of Official Analytical Chemists
BW: body weight
ADG: average daily gain
ADFI: average daily feed intake
F:G: feed to gain ratio
V/C: ratio of the villus height and crypt depth
*MUCIN-1*: *mucin-1*
*ITGB1*: *β 1-integrins*
*PKC*: *protein kinase C*
PCR: Polymerase Chain Reaction
OTUs: operational taxonomic units
PCoA: principal co-ordinates analysis
LDA: linear discriminant analysis
LEfSe: linear discriminant analysis effect size
eggNOG: Evolutionary Genealogy of Genes: Non-supervised Orthologous Groups
KEGG: Kyoto Encyclopedia of Genes and Genomes
KW: Kruskal-Wallis
COG: Cluster of Orthologous Groups of proteins
PICRUSt: phylogenetic investigation of communities by reconstruction of unobserved states
SCFAs: short-chain fatty acids.
